# Asymmetry Analysis of the Resonance Curve in Resonant Integrated Optical Gyroscopes

**DOI:** 10.3390/s19153305

**Published:** 2019-07-27

**Authors:** Yu Ming He, Fu Hua Yang, Wei Yan, Wei Hua Han, Zhao Feng Li

**Affiliations:** 1 Engineering Research Center for Semiconductor Integrated Technology, Institute of Semiconductors, Chinese Academy of Sciences, Beijing 100083, China; 2 Center of Materials Science and Optoelectronics Engineering, University of Chinese Academy of Sciences, Beijing 100049, China; 3 The School of Microelectronics, University of Chinese Academy of Sciences, Beijing 100049, China; 4 School of Electronic, Electrical and Communication Engineering, University of Chinese Academy of Sciences, Beijing 100049, China; 5 State Key Laboratory for Superlattices and Microstructures, Insititue of Semiconductors, Chinese Academy of Science, Beijing 100083, China

**Keywords:** gyroscopes, sensor, waveguide ring resonator, integrated optics

## Abstract

The Resonant Integrated Optic Gyroscope (RIOG) is a type of high accuracy gyroscope based on the Sagnac effect. A symmetrical resonance curve is very important to the performance of the RIOG. To further investigate and design a RIOG with a waveguide ring resonator, an in-depth research of the asymmetric resonance curve and its influence on the RIOG is fully developed. Four possible optical noises inducing the resonance curve asymmetry are analyzed and their mathematic models are established. These four optical noises are the normal mode effect, the backscattering noise, the backreflection noise and the polarization noise. Any asymmetry of the resonance curve will not only induce a large output bias error into the gyro output, but also seriously decrease the frequency discrimination parameter of the demodulation curve. By using a tunable fiber laser, the high aspect ratio silicon nitride WRR and the silicon dioxide WRR were tested. The experiment measured resonance curves can be well fitted with the theoretical simulation results. The experimental results show that a high aspect ratio silicon nitride waveguide can effectively suppress the polarization noise in the RIOG.

## 1. Introduction

Optical gyros based on the Sagnac effect are attractive sensors for inertial navigation systems [1,2,3,4]. However, both the Fiber Optic Gyroscope (FOG) and Ring Laser Gyroscope (RLG) have some serious disadvantages, as they are bulky, expensive, and energy intensive [5,6,7,8]. In order to overcome these shortcomings in traditional optical gyroscopes, the Micro Optic Gyroscope (MOG), which includes the Resonant Integrated Optic Gyroscope (RIOG) and the Interferometric Integrated Optical Gyroscope (IIOG), based on a waveguide ring resonator (WRR) was proposed [9,10,11,12,13,14,15,16]. The interferometric optical gyroscope is restricted by the transmission loss of the waveguide. Therefore, the RIOG is the most promising MOG to realize high accuracy. The RIOG uses a planar optical waveguide ring resonator instead of the fiber ring resonator to detect the Sagnac effect. Ultimately, it is expected that all optoelectronic devices will be integrated on one substrate [17,18].

Up till now, the transmission loss of the waveguide is still thousands times larger than that of the fiber. The internal defects of the waveguide are more serious, which will seriously affect the bias stability of the gyroscope and the linearity of its dynamic response. To our knowledge, the best long term bias stability of the RIOG have been reported is 15.12 °/h, which is still far from the requirements of aerospace and defence application requiring long term bias stability <10 °/h [19]. The key to further improve the performance of the RIOG is to suppress the optical noise. Several noise sources including the normal mode effect, the backreflection noise, the backscattering noise, and the polarization noise need to be fully researched and suppressed [20,21,22,23].

In this paper, the mathematic model of the RIOG base on a sinusoidal wave phase modulation spectroscopy technique is established. To quantitatively characterize the asymmetry of the resonance curve, a parameter of resonance asymmetry degree is defined. Four optical noises models were established, including the normal mode effect, the backscattering noise, the backreflection noise and the polarization noise. On the basis of the theoretical simulation results, corresponding recommendations are given.

## 2. System Structure of the Resonant Integrated Optical Gyroscope

### 2.1. The RIOG System Based on the Phase Modulation Spectroscopy Technique

RIOG is a high precision angular velocity sensor based on the Sagnac effect [24]. According to the Sagnac effect, the resonance frequency difference between the clockwise (CW) lightwave and the counterclockwise (CCW) lightwave is proportional to the rotational angular velocity. However, the frequency difference caused by rotation is very small. When the diameter of the ring resonator is 1 cm, in order to achieve a resolution of 1 °/h, the RIOG needs to detect the frequency difference of 0.002 Hz. Therefore, the signal detection system is critical to the performance of the RIOG and directly determines the sensitivity limit.

**Figure 1:**
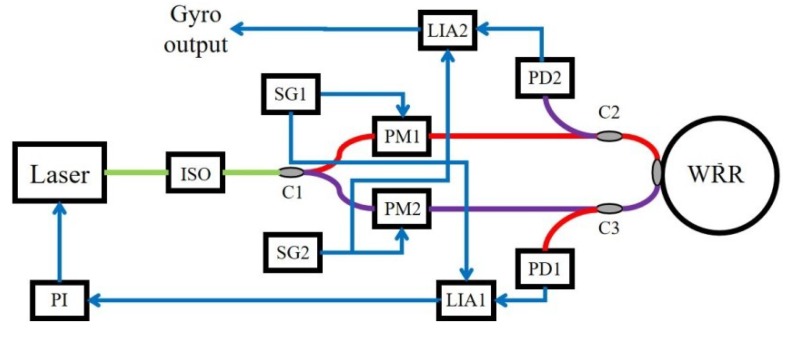
Conceptual diagram of the RIOG based on a phase modulation technique with a reflective resonator.

The signal detection system of the RIOG is usually based on the phase modulation and synchronous demodulation technique (PMST), which converts the resonant frequency difference into an optical intensity difference [25,26]. The system configuration of the RIOG is shown in Figure 1. The light emitted from a tunable narrow linewidth fiber laser is equally divided by a 3 dB coupler C1 and injected into a WRR in CW and CCW directions. Two phase modulators PM1 and PM2 are driven by the sinusoidal waveform from signal generators SG1 and SG2 with the modulation frequencies $f_{1}$ and $f_{2}$, respectively. The emergent light from the CCW lightwave is detected by the PIN photodetector (PD) PD1. The output of the PD1 is synchronously demodulated through the lock-in amplifier LIA1, and the demodulation signal is fed back to the proportional integrator (PI) to lock the laser frequency at the resonant frequency of the CCW lightwave. Therefore, the demodulation output of the LIA2 is proportional to the angular velocity and the rotation rate can be read out by digital operation.

### 2.2. The Resonance Curve and Demodulation Curve of the RIOG

In this paper, the RIOG system is based on a reflective resonator. A straight waveguide and a ring waveguide are placed together to form the directional coupler. The signal lights are injected from both ends of the straight waveguide. When the optical frequency of the incident light is equal to the resonant frequency of the WRR, periodic resonance dips will occur on the transmission curve. Therefore, the transmission curve of the WRR is also called resonance curve. The resonance curve of the WRR derived by multi-beam interference superposition method is as follows:

(1) $$T=\frac{{\left|E_{out}\right|}^{2}}{E_{0}^{2}}=P^{2}+\frac{R^{2}-2PR\left(cos\varpi \tau -V\right)}{1+V^{2}-2Vcos\varpi \tau }$$

(2) $$P=\sqrt{1-k_{c}}\sqrt{1\;-\;\alpha_{c}}$$

(3) $$R=k_{c}\left(1\;-\;\alpha_{c}\right)\sqrt{1\;-\;\alpha_{L}}$$

(4) $$V=\sqrt{1\;-\;\alpha_{L}}\sqrt{1-k_{c}}\sqrt{1-\alpha_{c}}$$

where ϖ is the mean optical angular frequency. τ and $\alpha_{L}\;$are the transmission time and the propagation loss of the beam transmitted around the ring waveguide for a round trip. τ=nL/c. L is the ring length of the WRR closed loop. $k_{c}$ and $\alpha_{c}\;$are the coupling coefficient and the insertion loss of the coupler. Several kinds of signal waves are researched and chosen to drive the phase modulator in a RIOG base on the PMST, which include sinusoidal wave, triangular wave, sawtooth wave, trapezoidal wave and hybrid wave [27,28,29,30]. The sawtooth wave phase modulation technique can effectively suppress backscattering noise, but it also brings a reset pulse error [31,32]. The triangular wave can avoid the influence of the reset pulse error. Meanwhile, considering the phase delay of each optical field components in the resonator, a transient optical field component is generated at the turning point of the triangular wave, which makes the output signals have transient response such as overshoot. Both digital triangular and sawtooth wave modulation techniques have a same problem that the duration of the digital step is limited by the relaxation time of the WRR. Trapezoidal wave and hybrid wave phase modulation techniques are relatively complex. Therefore, in this article, the analysis is based on the sinusoidal wave phase modulation technique.

**Figure 2:**
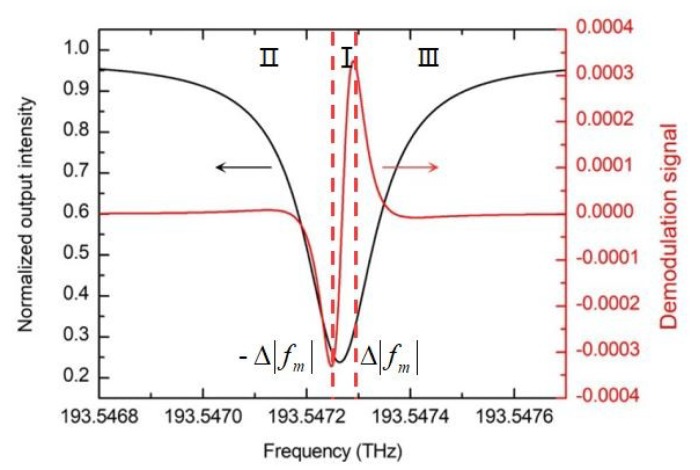
Schematic diagrams of the resonance curve and the demodulation curve. In this picture, the perimeter of the WRR is set to be 10.05 cm and the propagation loss is set to be 0.15. The coupling coefficient is 0.12 and the insertion loss of the coupler is 0.02 dB. The modulation frequency of the sinusoidal wave modulated signal is 2 MHz. The gain coefficient of the demodulation circuit is 0.7, and the photoelectric conversion efficiency is 1000. To achieve high carrier suppression, $J_{0}\left(M\right)=0$, the modulation index M is 2.405.

Assuming the fibers and waveguide are single mode transmission, all couplers and interfaces are considered lossless. Ignoring the influence of polarization, the complex amplitude of the beam after modulation can be expressed as a Bessel function expansion, as follows:

(5) $$E_{cw\_in}=\frac{1}{2}E_{0}\overset{\infty }{\underset{n=-\infty }{\sum }}J_{n}\left(M\right)e^{i2\pi \left(f_{0}+nf_{1}\right)t}$$

where $J_{n}$ is the Bessel function of order n. Suppose the amplitude and frequency of the sinusoidal modulated signal are $V_{1}$ and $f_{1}$, respectively. M and $V_{\pi }$ are the modulation index and half wave voltage of the phase modulator, $M=V_{1}\pi /V_{\pi }$. The complex amplitude of the resonant signal can be expressed as:

(6) $$E_{out}=\frac{1}{2}E_{0}\overset{\infty }{\underset{n=-\infty }{\sum }}J_{n}\left(M\right)e^{i2\pi \left(f_{0}+nf_{1}\right)t}h_{n}e^{i\varphi_{n}}$$

(7) $$h_{n}=\sqrt{P^{2}+\frac{2PVR+R^{2}-2PRcos\left(2\left(f_{0}+nf_{1}\right)\pi \tau \right)}{1+V^{2}-2Vcos\left(2\left(f_{0}+nf_{1}\right)\pi \tau \right)}}$$

(8) $$\varphi_{n}=arctan\left[\frac{Rsin\left(2(f_{0}+nf_{1})\pi \tau \right)}{P+PV^{2}+VR-\left(2PV+R\right)\cos\left(2\left(f_{0}+nf_{1}\right)\pi \tau \right)}\right]$$

where $h_{n}$ and $\varphi_{n}$ are the amplitude transfer coefficient and phase transfer coefficient of the WRR. The output voltage of the photodetector can be expressed as:

(9) $$V_{PD-out}=\frac{1}{8}NE_{0}^{2}\overset{\infty }{\underset{n=-\infty }{\sum }}\overset{\infty }{\underset{n^{\prime }=-\infty }{\sum }}J_{n}\left(M\right)J_{n^{\prime }}\left(M\right)e^{2\pi }e^{i2\pi f_{1}t\left(n-n^{\prime }\right)}h_{n}h_{n^{\prime }}e^{i\left(\varphi_{n}-\varphi_{n^{\prime }}\right)}$$

where N is the photoelectric conversion efficiency. Since the LIA demodulation is base on the first harmonic, the gyroscope ouput signal after synchronous demodulation can be expressed as $V_{LIA-out}=\frac{1}{8}NE_{0}^{2}G\underset{n}{\sum }J_{n}J_{n+1}h_{n}h_{n+!}sin\left(\varphi_{n+1}-\varphi_{n}\right)$. Considering that $J_{2n+!}\left(M\right)=-J_{-\left(2n+!\right)}\left(M\right)$ and $J_{2n}\left(M\right)=J_{-2n}\left(M\right)$, the corresponding expression of the demodulation curve can be expressed as:

(10) $$V_{LIA-out}=\frac{1}{8}NE_{0}^{2}G\overset{\infty }{\underset{n=0}{\sum }}J_{n}J_{n+1}\left\{h_{n}h_{n+1}sin\left(\varphi_{n+1}-\varphi_{n}\right)-h_{-n}h_{-n-1}sin\left(\varphi_{-n}-\varphi_{-n-1}\right)\right\}$$

where G is the gain coefficient of the demodulation circuit. Figure 2 shows the calculation results of the transmission output and the demodulation output under the experimental conditions using a silicon nitride WRR. $\pm \left|\Delta f_{m}\right|$ is the resonance frequency deviation when the demodulation curve is at the extreme value. $dI_{LIA-out}/d\Delta f=0$. The demodulation curve of the gyroscope can be divided into two regions, the middle linear region (region I) and the decreasing function region (region II and region III). As shown in Figure 2, in region I, the demodulation output is approximately proportional to the frequency deviation. Therefore, the RIOG system can use this curve to track the resonance frequency of CCW lightwave and output the rotation signal of CW lightwave. The slope of the linear region determines the ultimate sensitivity of the gyroscope, which is called frequency discrimination parameter. $\Delta f_{FWHM}$ is the full width at the half maximum (FWHM) of the resonant curve. The working range of the RIOG is about $\Delta f_{FWHM}/\sqrt{3}$. The frequency corresponding to the zero point in the linear region is the resonant frequency, that is, when the laser frequency equals the resonant frequency, the output of the demodulated signal is zero.

**Figure 3:**
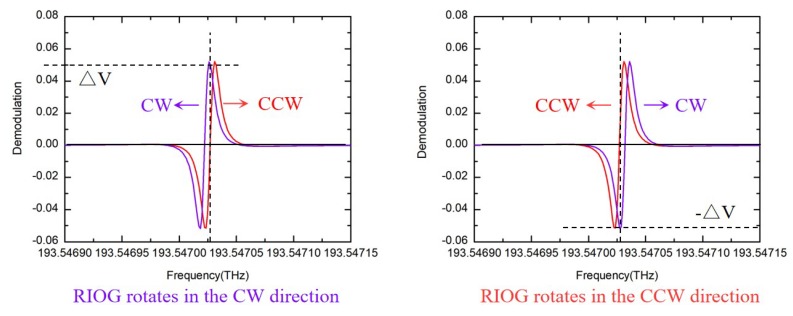
The principle of the rotational angular velocity signal detection.

The RIOG detection principle can be expressed more intuitively with the demodulation curve. As shown in Figure 3, the laser frequency is fixed on the resonant frequency of the CCW lightwave. The magnitude of the CW lightwave demodulation signal ΔV is proportional to the rotational angular velocity. The sign of the ΔV reflects the gyroscope rotation direction. The demodulation signal is only related to the frequency discrimination parameter and the rotational angular velocity. Therefore, when the frequency discrimination parameter of the gyro is given, the rotational angular velocity can be calculated from the demodulation signal.

## 3. Analysis Asymmetry of the Resonance Curve

**Figure 4:**
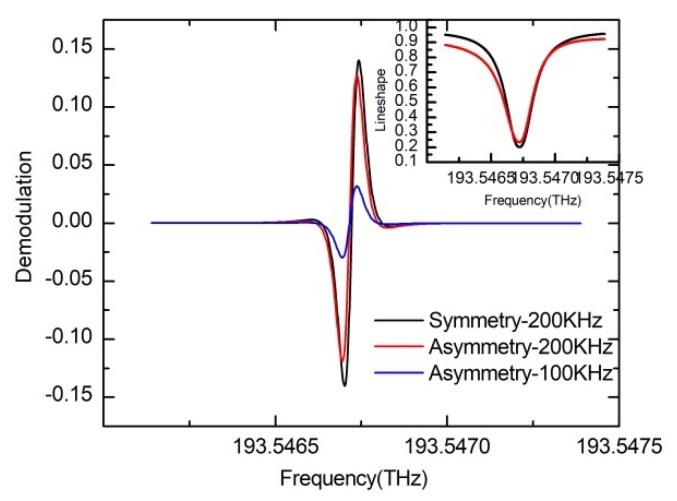
The asymmetry resonance curves and demodulation curve with different modulation frequencies.

In this section, the noise factors causing the transfer curve asymmetry are discussed in detail. The measured resonance curves are fitted to confirm our analysis. When the resonance curve is asymmetric, the zero point of the demodulation curve deviates from the resonance frequency point, and changes slightly with the external environment. In RIOG systems, the backscattering noise is reduced by carrier suppression and different modulation frequencies in the CW and CCW lightwaves [33]. When different modulation frequencies are applied, the zero points of the demodulation curves are also different, as shown in Figure 4. Therefore, the demodulation output of the CW lightwave is not zero [34,35]. The above analysis is based on the premise that the resonance curves of the CW and CCW lightwaves are the same. If the resonance curves are not only asymmetric but also different, the asymmetric resonance curve will cause more deviations. Additionally, we can find that the frequency discrimination parameter of the demodulation curve will be reduced due to the asymmetry.

### 3.1. Materials and Methods

**Figure 5:**
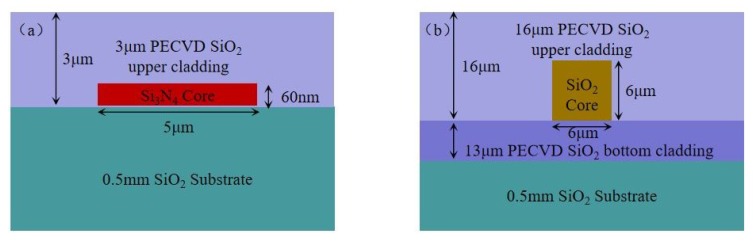
Cross sections of the (**a**) silicon nitride waveguide and the (**b**) silicon dioxide waveguide.

In order to realize a high sensitivity gyroscope, we fabricated the WRR base on a silicon nitride waveguide and a silicon dioxide waveguide. The structure diagram of the silicon nitride waveguide and silicon dioxide waveguide are shown in Figure 5a,b, respectively. The refractive index of the Si_3_N_4_ core layer is 1.97. Low-pressure chemical vapor deposition (LPCVD) is used to deposit about 60-nm-thick Si_3_N_4_ film on the silicon dioxide substrate. Reactive ion etching (RIE) is used to etch through the Si_3_N_4_ film, thus defining the waveguide core widths. Plasma enhanced chemical vapor deposition (PECVD) is used to deposit about 3 μm silicon dioxide cladding layer ($n_{sio_{2}}=1.457$). The refractive index of the cladding layer is equal to that of the substrate by doping boron and phosphorus. The effective refractive index of the silicon nitride waveguide is 1.468. For the silicon dioxide waveguide, the index contrast is around 0.7% ($n_{core}=1.456,n_{clad}=1.445$). The effective refractive index is 1.451. The coupling coefficients of the silicon nitride WRR and the silicon dioxide WRR are designed to be 0.12 and 0.15, respectively. The perimeters of the silicon nitride WRR and the silicon dioxide WRR are 5.4 cm and 10.05 cm, respectively. To compare the influences of different noises on the gyro output, the relevant parameters are set to be the same. The WRR perimeter and the effective refractive index are set to be 10.05 cm and 1.468. The coupling coefficient and the insertion loss are set to be 0.12 and 0.02 dB, respectively. The WRR propagation loss is set to be 0.15 dB. The modulation frequency is 2 MHz.

**Figure 6:**
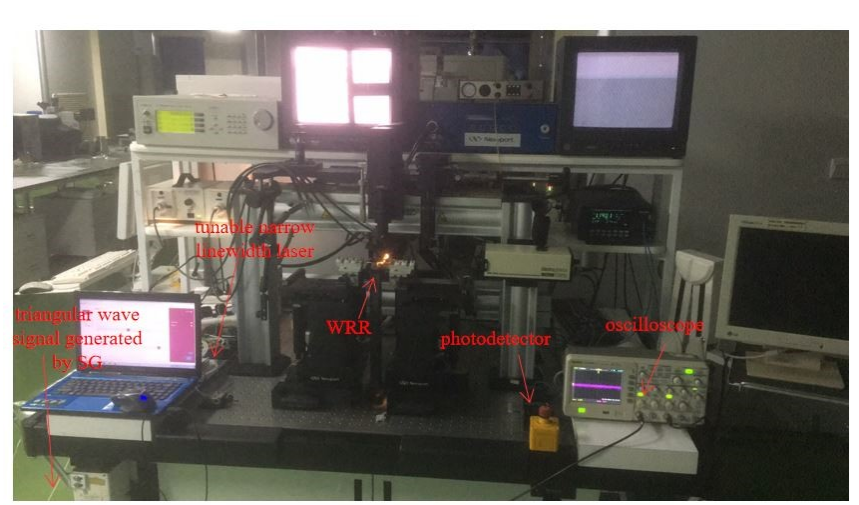
The measuring instrument of the WRR.

The most effective way to analyze the WRR is to get its resonance lineshape. As shown in Figure 6, a tunable narrow linewidth fiber laser (Koheras BASIK E15, NKT Photonics, Compenhagen, Denmark) is used. The linewidth (Lorenzian) of the laser is less than 0.1 kHz. The laser frequency is tuned at 1549 nm–1551 nm by a triangular wave signal of 5 V and 100 Hz. Fiber optics and the WRR are aligned precisely by means of a coupling system. Finally, the output light of the WRR is detected by a PD and presented on an oscilloscope.

### 3.2. Resonance Asymmetry Degree

In order to quantitatively characterize the asymmetric degree of the resonance curve, we introduce the concept of resonance asymmetry degree. The resonance asymmetry degree is defined as [34]:

(11) $$k_{RAR}=\frac{W_{a}-W_{b}}{W_{a}+W_{b}}=\frac{W_{a}-W_{b}}{\Delta f_{FWHM}}$$

**Figure 7:**
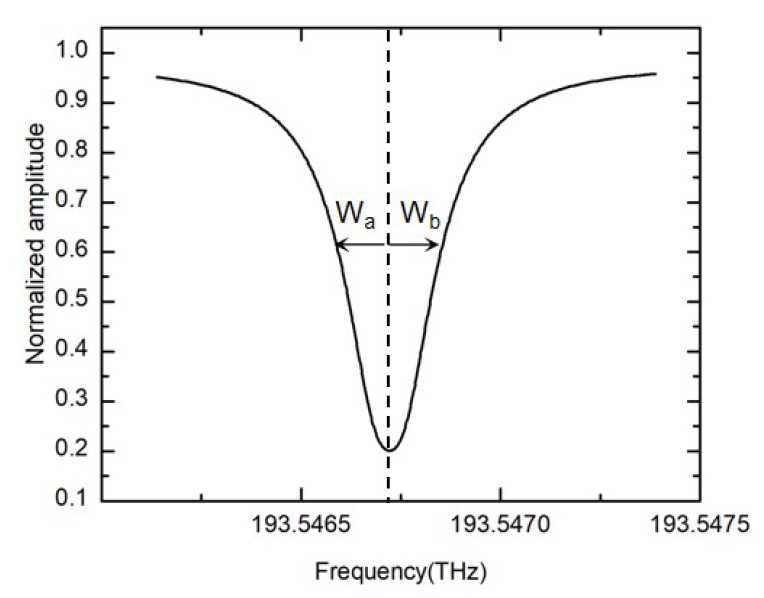
The definition of the resonance asymmetry degree.

The coefficient $k_{RAR}$ is defined as the asymmetry at the full width at half maximum (FWHM) of the resonance curve for convenience. Where $W_{a}$ is the half width at the half maximum at the left side with respect to the resonance dip, and $W_{b}$ is the half width at the half maximum at the right side with respect to the resonance dip. Figure 7 shows a symmetrical resonance curve. When resonance curve is symmetrical, the resonance asymmetry degree is equal to zero.

### 3.3. Normal Mode Effect

**Figure 8:**
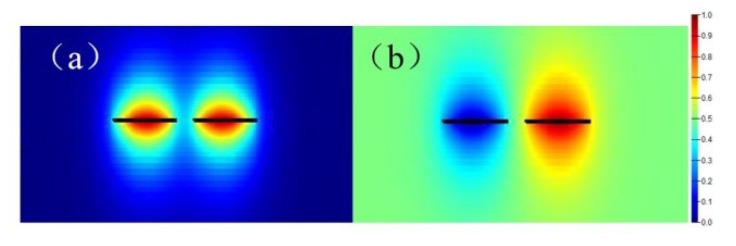
(**a**) The symmetric mode filed and (**b**) the antisymmetric mode filed.

In an evanescent field directional coupler, there are generally existing two normal modes, a symmetric mode and an antisymmetric mode [21]. The field distribution of the symmetric mode is closer to the inner side of the coupler, while that of the antisymmetric mode is closer to the outside. Taking a silicon nitride WRR as an example, and the coupling space is 1.5 μm. The normal modes distribution can be calculated by Lumerical Mode Solution, as shown in Figure 8a,b. The effective refractive index of the symmetric mode and the antisymmetric mode are 1.468 and 1.467, respectively.

For an ideal evanescent field directional coupler, the insertion losses of the two normal modes are equal. However, due to the influence of impurities or defects near the coupler region, the losses of the two normal modes are different. Considering the differential normal mode losses, the coupling ratio of the coupler is rewritten as:

(12) $$k_{c}^{’}={\left(\frac{\rho_{+}-\rho_{-}}{2}\right)}^{2}+\rho_{+}\rho_{-}sin^{2}\frac{\pi L_{co}\Delta n}{\lambda }$$

where $\rho_{+}$ and $\rho_{-}$ are the amplitude transfer ratio of the symmetric mode and the antisymmetric mode, respectively. Δn is the effective refractive index difference between two normal modes. λ is the wavelength of the laser. $L_{co}$ is the length of the coupling region. Considering the normal mode effect, the relative phase difference between the lights in the two waveguides is:

(13) $$\gamma =arctan\left(\frac{2\rho_{+}\rho_{-}sin(2\pi L_{co}\Delta n/\lambda )}{\rho_{+}^{2}-\rho_{-}^{2}}\right)$$

Due to the normal mode effect, the relative phase difference deviates from the lossless value π/2 [21]. Combining Equations (1), (12) and (13), the normalized transmission spectrum of the WRR can be expressed as:

(14) $$T_{s}=t_{1}t_{2}{\left(P^{\prime }+R^{\prime }e^{2i\gamma }\frac{1}{e^{i\omega \tau }-V^{\prime }}\right)}^{2}$$

where:

(15) $$P^{\prime }=\sqrt{1-k_{c}^{’}}\sqrt{1-\alpha_{c}}$$

(16) $$R^{\prime }=k_{c}^{’}\left(1-\alpha_{c}\right)\sqrt{1-\alpha_{L}}$$

(17) $$V^{\prime }=\sqrt{1-\alpha_{L}}\sqrt{1-k_{c}^{’}}\sqrt{1-\alpha_{c}}$$

**Figure 9:**
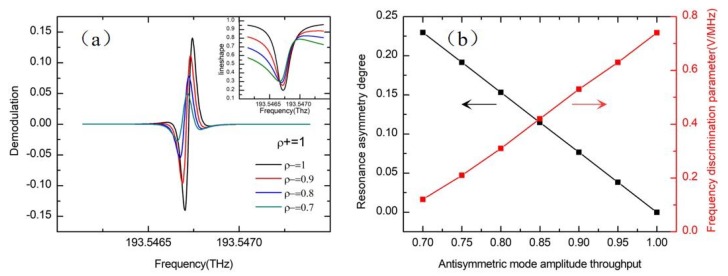
(**a**) The influence of the normal mode effect on the resonance curve and the demodulation curve. (**b**) Effect of the normal mode on the resonance asymmetry degree and the frequency discrimination parameter of the gyroscope. The modulation frequency is 2 MHz.

Figure 9a shows the simulation results of the transmission curve and the demodulation curve considering the normal mode effect. The WRR is demodulated by a 2 MHz sinusoidal wave. The resonance asymmetry degree is proportional to the amplitude throughput difference between two normal modes. In particular, when $\rho_{+}$ = $\rho_{-}$, the resonance curve is symmetrical. As shown in Figure 9a, the zero points of the demodulation curve change with the normal modes loss difference, and a bias error is produced. Besides, the frequency discrimination parameter of the demodulation curve will be reduced due to the asymmetry. Assuming the symmetric mode amplitude throughput is $\rho_{+}=1$, Figure 9b is obtained while the antisymmetric mode throughput varies. It shows that the resonance asymmetry degree linearly increases by increasing the two normal modes loss difference, and due to the asymmetry resonance curve, the frequency discrimination coefficient linearly decreases. As the nomal mode loss difference increases from 0 to 0.3, the frequency discrimination parameter decreases linearly from 0.74 V/MHz to 0.12 V/MHz about six times. When the antisymmetric mode throughput is $\rho_{-}=0.9$, the gyro bias deviation is calculated to be as high as 1.28 rad/s.

**Figure 10:**
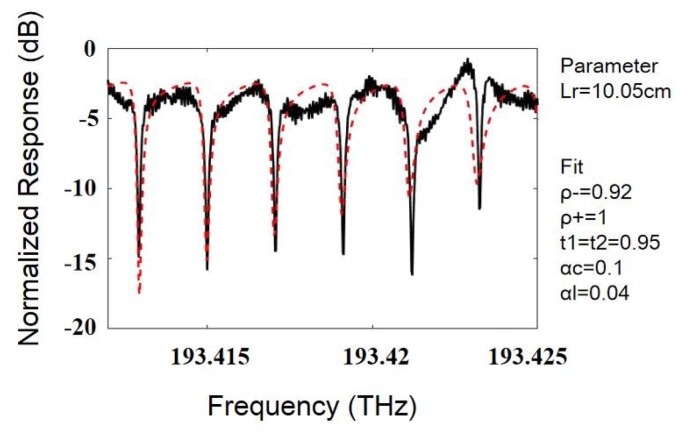
Measured resonance curve and the fitting results of the silicon dioxide WRR.

Figure 10 shows the measured resonance curve of a silicon dioxide WRR. The free spectral range (FSR) of the measured resonance curve is 2.1 GHz. The finesse and the resonance depth are 8.7 and 0.9, respectively. The quality factor is 8.1×10^5^. Base on the silicon dioxide WRR, the sensitivity limit of the RIOG is 9.07 deg/h ($\delta \Omega =\frac{\lambda c}{2FA\rho }\sqrt{\frac{hfB}{\eta T_{max}}}$, assuming the maximum optical power received by photodetector is $T_{max}=1\;\mathrm{mW}$, the quantum efficiency of the PD is η=0.9, the system bandwidth is B=1Hz). The resonance asymmetry degree was obtained through fitting. The resonance asymmetry degree is −0.133, and the gyro bias deviation is 2.09 deg/s. One possible reason is that an impurity particle inside the coupler region, which results in the amplitude throughput of the symmetric mode much less than the antisymmetric mode. From the above analysis, it can be concluded that the normal mode effect can lead to strong asymmetry of the resonance curve. Therefore, it is necessary to suppress the normal mode effect. Under ideal conditions, the coupler in the WRR is an isotropic device, that is, the asymmetry of clockwise and counterclockwise light should be the same. The zero offset can be reduced by reducing the modulation frequency difference, but the frequency discrimination parameter cannot be improved by this method. Some articles proposed to use a transmissive ring resonator instead of a reflective ring resonator in order to avoid the normal mode effect [35]. However, the transmissive waveguide ring resonator is still limited by the large transmission loss of the waveguide. It is possible to solve this problem by further controlling contaminants and reducing waveguide sidewall scattering loss by using a high aspect ratio waveguide.

### 3.4. Rayleigh Backscattering Noise

Rayleigh backscattering noise is one of the main optical noises in the RIOG. During the waveguide manufacturing process, the material density distribution is non-uniform, which leads to an uneven refractive index distribution. This inhomogeneity will cause light scattering, called Rayleigh backscattering [7,36,37]. The energy of the Rayleigh scattering is proportional to the reciprocal of the fourth power of the wavelength. Assuming that the waveguide is single mode, ignoring the birefringence effect, and an infinite coherence length of the laser, the total light intensity detected by the photodetector can be expressed as:

(18) $$I=I_{1}+I_{2}+I_{3}$$

where $I_{1}$, $I_{2}$, and $I_{3}$ represent the intensity of the signal light, the interference light and the backscattering light, respectively. Considering the signal light intensity detected by the detector is a Lorentzian spectral line shape, the backscattering light intensity can be expressed as:

(19) $$I_{3}=\frac{\alpha_{R}SL*k_{c}^{2}\left(1-\alpha_{c}\right)}{{\left(e^{i\varpi \tau }-V\right)}^{4}}*E_{0}^{2}$$

where $\alpha_{R}$ is the Rayleigh scattering coefficient [38]. The recapture factor is S, that is, $S=3/2n^{2}W_{0}^{2}{\left(\varpi /c\right)}^{2}$. Considering the refractive index fluctuation of the entire material has an ensemble average of zero 〈Δn〉=0 and $I_{2}$ is much larger than $I_{3}-\langle I_{3}\rangle$, so $I_{2}$ can be expressed as the variance of I:

(20) $$I_{2}=\frac{2e^{-L\alpha }E_{0}^{2}\sqrt{LS\alpha_{R}\left(P^{2}+{\left(PV+R\right)}^{2}-2P\left(PV+R\right)cos\left(\omega \tau \right)\right)k_{c}}}{{\left(1+V^{2}-2Vcos\left(\omega \tau \right)\right)}^{3/2}}cos\left(\varepsilon +\varepsilon_{0}\right)$$

ε and $\varepsilon_{0}$ are the cosine function phase constant and the thermal floating phase difference, respectively.

**Figure 11:**
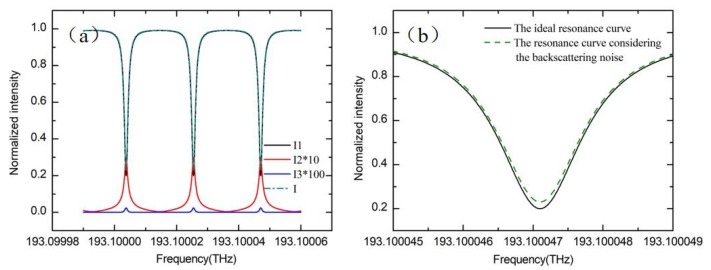
(**a**) Resonance characteristics of the $I_{1}$, $I_{2}$, and $I_{3}$ when gyro is static. (**b**) The influence of the backscattering noise on the resonance depth.

Assuming that the $\alpha_{R}=S=0.001$. Figure 11a shows the simulation result of the transmission curve when the gyroscope is static. The transmission curve is completely symmetrical because the resonance frequency of the backscattered light is exactly equal to the resonance frequency of the signal light. Figure 11b shows the influence of the backscattering noise on the resonant depth. When Rayleigh backscattering noise is introduced, the resonant depth of the resonant ring is reduced from 0.8 to 0.77. Meanwhile, the finesse of the cavity drops from 82.1 to 79.3, which will reduce the sensitivity of the RIOG.

**Figure 12:**
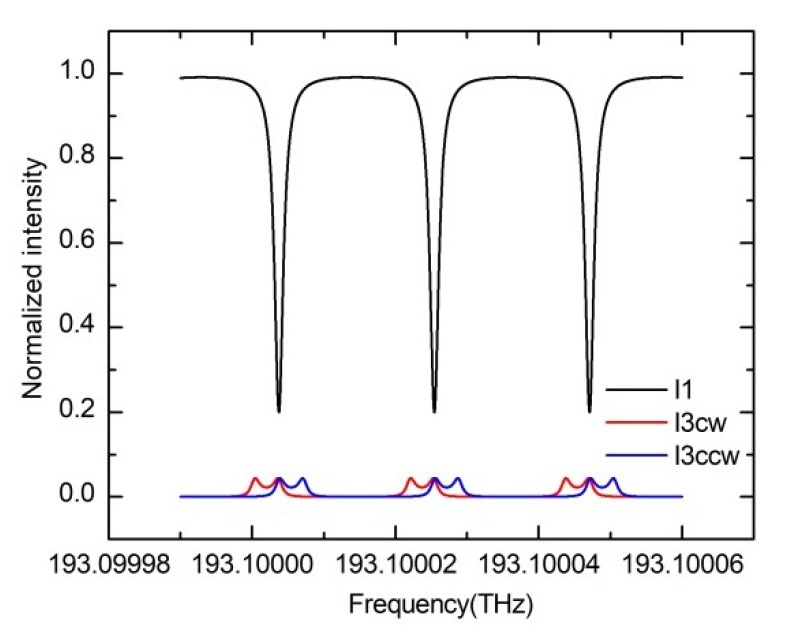
Calculated resonance curves when the Rayleigh scattering coefficient and the recapture factor are 0.001. The red solid line is the simulation result of the I_3_ when the gyroscope rotates CW. The blue solid line is the simulation result of the I_3_ when the gyroscope rotates CCW.

When the gyroscope rotates, the resonance curve considering the Rayleigh backscattering noise will split into two peaks. In this case, the backscattered light has both the resonance information of the CW and CCW lightwaves. The frequency difference between the two peaks is twice the Sagnac phase shift. As shown in Figure 12, the Sagnac phase shift caused by a rotation of θ=1 rad. The red solid line and the blue solid line are the resonance curve considering the Rayleigh backscattering noise when the gyro rotates at CW and CCW directions. When the gyro rotates, the transmission curve is asymmetrical, which affecting the gyro output. Phase modulation technique can effectively suppress the backscattering noise. By modulating the clockwise and counterclockwise lights with different frequencies, the backscattering noise can be filtered by the LIA. The LIA only demodulates the signal light with the corresponding frequency. For the interferometric optical noise, the intensity of the interference term can be reduced by carrier suppression. A phase difference traversal method can also efficiently suppress the influence of the interference light [39].

### 3.5. Backreflection Noise

**Figure 13:**
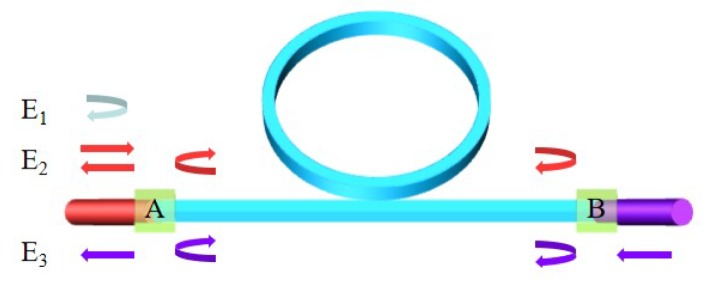
Conceptual diagram of the backreflection light in a reflective resonator.

The backreflection noise can also contribute to the resonance curve asymmetry [40,41]. As shown in Figure 13, the WRR is coupled to the fiber at point A and point B. Because of the endface backreflection, the light detected by the photodetector can be expressed as the sum of $E_{1}$, $E_{2}$, and $E_{3}$. $E_{1}$ represents the light which directly reflected back into the fiber at point A. $E_{2}$ represents the light which is injected into the waveguide at point A and finally reflected back to the fiber at point A. $E_{3}$ represents the light which is injected from the point B and then coupled into the fiber at point A.

**Figure 14:**
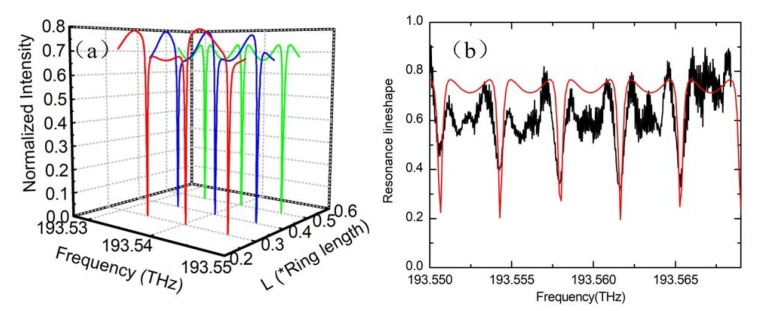
(**a**) Resonance characteristics of the intensity term $I_{3}$ with different straight waveguide length; (**b**) the measured resonance curve and the fitting results of the silicon nitride WRR.

Assuming the laser linewidth can be neglected, and there is no reflection point in the waveguide ring resonator. With the multi-beam interference superposition method, $E_{1}$,$\;E_{2}$,$\;E_{3}$ can be obtained as follows:

(21) $$E_{1}=\sqrt{r_{1}}E_{0}e^{i\left(\omega \tau +\phi_{0}\right)}$$

(22) $$E_{2}=t_{1}E_{0}e^{i\left(\omega \tau +\phi_{0}\right)}\overset{\infty }{\underset{N=1}{\sum }}r_{1}^{\left(N-1\right)/2}r_{2}^{N/2}e^{i2NkL}{\left(P+Re^{i\pi }\frac{1}{e^{i\varpi \tau }-V}\right)}^{2N}$$

(23) $$E_{3}=\sqrt{t_{1}t_{2}}E_{0}e^{i\varpi \tau }\overset{\infty }{\underset{N=1}{\sum }}{\left(r_{1}r_{2}\right)}^{N-1/2}{\left(Pe^{ikL}+Re^{i\left(kL+\pi \right)}\frac{1}{e^{i\varpi \tau }-V}\right)}^{2N-1}$$

where $r_{1},\;r_{2}$ and $t_{1}$, $t_{2}$ are the reflectance ratios and coupling efficiencies at the two A and B points. k is the wave number in vacuum. $\phi_{0}$ is the phase difference at the two A and B points. Considering the initial phase of the $E_{1}$ and $E_{2}$ are equal, the total light intensity detected by the photodetector can be expressed as:

(24) $$I=\frac{1}{2}\left(I_{1}+I_{2}+I_{3}\right)=\frac{1}{2}\left({\left|E_{1}+E_{2}\right|}^{2}+2Re\left[\left(E_{1}+E_{2}\right)E_{3}^{*}\right]+{\left|E_{3}\right|}^{2}\right)$$

where $I_{1}$, $I_{2}$, and $I_{3}$ are the intensity of the reflected light, the interference light and the signal light, respectively. On the basis of the analysis above on the backscattering noise, the noise introduced by $I_{1}$ and $I_{2}$ can be eliminated by the phase modulation technique. In this section, we will fully research the resonant characteristics of the third term $I_{3}$. When the gyro is stationary, Figure 14a shows the resonance characteristics of $I_{3}$ when the gyroscope is stationary. The reflectance ratios and the coupling ratios are $r_{1}=r_{2}=0.05,\;t_{1}=t_{2}=0.6$, respectively. The resonance asymmetry degree is related to the length of the straight waveguide. If the length of the straight waveguide is equal to half of the waveguide ring length, then the resonance curve is symmetrical, that is because of the resonance period of the Fabry-Perot cavity is equal to the FSR of the WRR. Otherwise, the resonance curve is asymmetrical. Figure 14b shows the measured resonance curve of the silicon nitride WRR. The red curve is the fitting result of the resonance curve considering the backreflection noise. The reflectance ratio is approximately 0.094. The lengths of the ring resonator and the straight waveguide are 5.4 cm and 2.7 cm. The FSR of the measured silicon nitride WRR is 3.6 GHz. The finesse and the resonance depth are 6.3 and 0.5, respectively. The quality factor of the silicon nitride WRR is $3.2\times 10^{5}$. The resonance notch is symmetric. Therefore, the backreflection noise effect can be reduced by setting the length of straight waveguide to be half the ring length.

**Figure 15:**
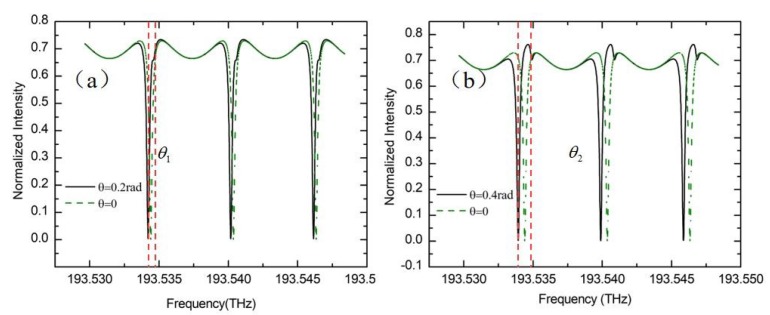
Considering the backreflection noise, (**a**) the resonance curve in the case of Sagnac phase shift is 0.2 rad, and (**b**) the resonance curve in the case of the Sagnac phase shift is 0.4 rad can be obtained.

When the gyroscope rotates, the $E_{3}$ needs to be rewritten as:

(25) $$\begin{array}{ll} E_{3}=\sqrt{t_{1}t_{2}}E_{0}e^{i\varpi \tau } & \sum_{N=2}^{\infty }{\left(r_{1}r_{2}\right)}^{N-1/2}[{\left(Pe^{ikL}+Re^{i\left(kL+\pi \right)}\frac{1}{e^{i\left(\varpi \tau -\theta \right)}-V}\right)}^{N-1}\\ & *{\left(Pe^{ikL}+Re^{i\left(kL+\pi \right)}\frac{1}{e^{i\left(\varpi \tau +\theta \right)}-V}\right)}^{N}+\left(Pe^{ikL}+Re^{i\left(kL+\pi \right)}\frac{1}{e^{i\left(\varpi \tau +\theta \right)}-V}\right)] \end{array}$$

where θ is the Sagnac phase shift caused by the rotation. Assuming the reflectance ratios and coupling efficiencies at the two A and B points are $r_{1}=r_{2}=0.1$, $t_{1}=t_{2}=0.6$, respectively. The straight waveguide length is set to be half of the ring length. The theoretical calculation results of I_3_ are shown in Figure 15a,b. The green dashed line is the resonance curve when the gyro is stationary. When the gyroscope is at rotating, the resonance peak will split up into one deep peak and one shallow peak. The frequency difference between these two resonance peaks is approximately equal to the Sagnac phase shift caused by the rotation. Also it should be noticed that the resonance asymmetry degree will change with the Sagnac phase shift slightly due to the interaction between the resonance curves of WRR and Fabry-perot cavity.

**Figure 16:**
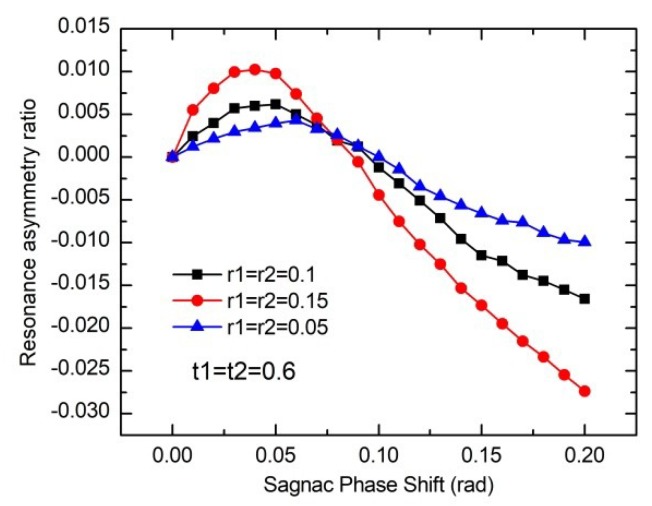
The influences of the Sagnac phase shift on the reonance asymmetry degree considering the backreflection noise.

In order to illustrate this effect more specifically, different Sagnac phase shifts of the WRR considering the backreflection noise are investigated, which is shown in Figure 16. When the gyroscope is at rest, the resonance frequencies of the two resonance peaks are exactly at the same point, and the resonance asymmetry degree of the resonance curve is zero. When the gyroscope rotates, the characteristic of $I_{3}$ with two resonance notches will induce the resonance curve asymmetry. With the increases of the resonance asymmetry degree, the working range and the sensitivity of the RIOG decrease. Therefore, besides relying on a sinusoidal wave phase modulation technique, we also need to reduce the end face reflection ratio to suppress the backreflection noise. The resonance asymmetry degree can be reduced by decreasing the reflectance ratio. In the packaging of the WRR with optical fibers, the waveguide end face and the fiber coupling end face should be obliquely polished to make the reflecting surface not perpendicular to the light propagation direction, so as to prevent the backreflection light from forming the guiding mode to continue to propagate in the waveguide [42].

### 3.6. Polarization Noise

Due to the effect of the birefringence, there are two orthogonal polarization states in the RIOG. The main polarization state is used to detect the rotation signal. However, the unwanted polarization state introduces the error. While the two resonance dips are in close proximity, changes resonance asymmetries are observed [43].

**Figure 17:**
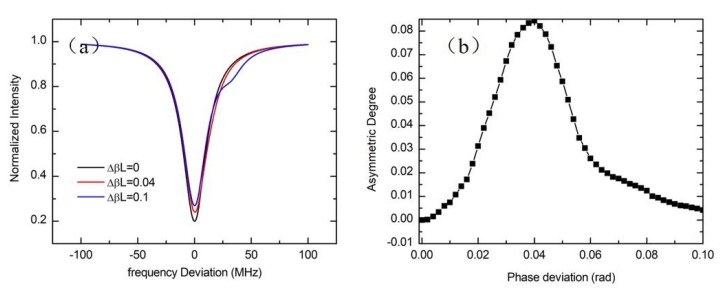
(**a**) Simulated resonance curves with different polarization phase deviation; (**b**) the influence of the phase deviation between two polarization states on the resonance asymmetry degree.

In this part, the influence of the polarization noise is theoretically analyzed. Assuming the polarization directions of the coupler input and output waveguides are parallel to each other. Meanwhile, the insertion losses and the transmission losses of the two polarization states are equal. By using the Jones matrix approach, the signal received by the PD can be express as [20]:

(26) $$I_{PD2}=\frac{1}{2}\left\{\left(1-K\right)\left[1-\rho \Gamma \left(f\right)\right]+K\left[1-\rho \Gamma \left(f-\frac{\Delta \beta L}{2\pi \tau }\right)\right]\right\}I_{0}$$

(27) $$\Gamma \left(f\right)=\frac{\Delta f_{FWHM}^{2}}{\Delta f_{FWHM}^{2}+4{\left(f-f_{0}\right)}^{2}}$$

where K is the excitation coefficient of the unwanted polarization state, and ΔβL is the phase deviation of the two polarization states transmitted in the ring for a round trip. τ is the traveling time in the WRR. f is the laser frequency. ρ and $f_{0}$ are the resonance depth and the resonance frequency of the resonance curve. The FWHM and the resonance depth are set to be 25.3 MHz and 0.8 according to the theoretical calculation results of the simulation WRR structure. The excitation ratio of the unwanted polarization state is 0.1. Figure 17a shows the influences of ΔβL on the resonance asymmetry degree of the resonance curve. The phase deviation of the two polarization states are 0, 0.04, and 0.1, respectively. Due to the s interaction between the two polarization states, the resonance curve will be asymmetry. The resonance asymmetry degree depends on the resonance frequency difference and the depth of the two polarization states. The resonance frequency difference determined by the propagation constant difference between two polarization states, which varies with the external environment. Meanwhile, the depth of the unwanted resonance peak depends on the excitation coefficient of the unwanted polarization state. As shown in Figure 17b, the resonance asymmetry degree reaches the maximum value of 0.084 with the phase deviation is close to the half-width of the FWHM. $\Delta \beta L\approx \pi \tau \Delta f_{FWHM}$.

**Figure 18:**
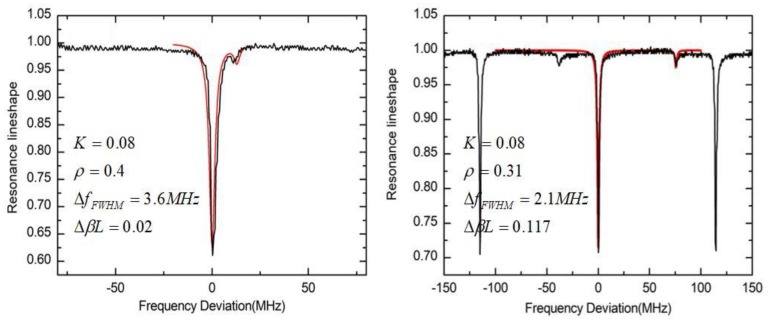
The measured resonance curve and the fitting results of the silicon dioxide WRR.

Figure 18 shows the measured resonance curve of the silicon dioxide WRR. The resonance asymmetry degrees of the silicon dioxide WRR are −0.05 and −0.02, respectively. The phase deviation of the two polarization states are 0.02 and 0.117. According to the result of Figure 17b, the polarization noise can be reduced by separating the peaks of the two polarization states. Therefore, by controlling the environment temperature, the asymmetry caused by polarization noise can be reduced. This method is to make the resonance peaks of the two polarization states as staggered as possible, that is, the phase difference between the two polarization states is π/2 [44]. However, it is very difficult to accuracy control the environment temperature and does not meet the requirements for integration. Therefore, it is necessary to reduce the excitation ratio of the unwanted polarization sate. An efficient method is to design and use a waveguide structure with high suppression ratio of the unwanted polarization state. The silicon nitride WRR based on a high aspect ratio waveguide can effectively suppress the sub-polarization state, and no obvious polarization noise is observed in the silicon nitride WRR experiment.

## 4. Conclusions

In summary, we have briefly introduced a RIOG system based on the phase modulation technique. The normal mode effect, the backreflection noise, the backscattering noise and the polarization noise in the RIOG are theoretically analyzed, and their mathematic models are established. Besides, the influences of these noises on the resonance asymmetry degree are fully researched. In the RIOG system, an asymmetric resonance curve not only induces a bias error into the gyro output but also decreases the frequency discrimination coefficient.

Considering these noises, we suggest that the waveguide structure of the WRR adopt a high aspect ratio silicon nitride waveguide. On the one hand, this can suppress the unwanted polarization state to reduce the polarization noise. On the other hand, this low loss waveguide structure is conducive to improving the sensitivity limit of the RIOG. The RIOG system with different frequency modulation signals and precise modulation coefficient can effectively suppress the backscattering noise and backreflection noise. It should be noted that due to the interaction between the resonance curves of Fabry Perot cavity and WRR, the degree of resonance asymmetry of the resonance curve slightly changes with the rotation angular velocity. It is recommended to suppress the formation of backreflected light by obliquely polishing the waveguide end face. These results would provide important reference for the research on the RIOG and the waveguide ring resonator.
